# Retraction: The Effect of Social Stress on Chronic Pain Perception in Female and Male Mice

**DOI:** 10.1371/journal.pone.0156567

**Published:** 2016-05-25

**Authors:** The PLOS ONE Editors

It has come to the attention of the PLOS ONE Editors that there are substantial overlaps between the text of this article and a number of previously published works by other authors, particularly in the Introduction and Discussion sections. Text was duplicated verbatim or with minor modifications from the sources listed below:

Farrell C, McAvoy H, Wilde J (2008) Tackling health inequalities: An all-Ireland approach to social determinants. Islandbridge, Dublin: Combat Poverty Agency Publication. (Reference 1 in the published article) [2]

Vendruscolo LF, Pamplona FA, Takahashi RN (2004) Strain and sex differences in the expression of nociceptive behavior and stress-induced analgesia in rats. Brain Res 1030: 277–283. doi: 10.1016/j.brainres.2004.10.016 (Reference 5 in the published article) [3]

Racine M, Tousignant-Laflamme Y, Kloda LA, Dion D, Dupuis G, et al. (2012) A systematic literature review of 10 years of research on sex/gender and pain perception–Part 2: Do biopsychosocial factors alter pain sensitivity differently in women and men? Pain 153: 619–35. doi: 10.1016/j.pain.2011.11.026 (Reference 17 in the published article) [4]

Sex differences in opioid-mediated pain inhibitory mechanisms during the interphase in the formalin test. 2007. Neuroscience. Volume 146, Issue 1, 25 April 2007, Pages 366–374 (uncited in the published article) [5]

The formalin test: a dose–response analysis at three developmental stages. 1998. Pain Volume 76, Issue 3, June 1998, Pages 337–347 (uncited in the published article) [6]

Glynn P, Coakley R, Kilgallen I, Murphy N, O'Neill S (1999) Circulating interleukin 6 and interleukin 10 in community acquired pneumonia. Thorax 54: 51–55. doi: 10.1136/thx.54.1.51 (Reference 35 in the published article) [7]

Gioiosa L, Chiarotti F, Alleva E, Laviola G (2009) A trouble shared is a trouble halved: social context and status affect pain in mouse dyads. PloS one 4: e4143. doi: 10.1371/journal.pone.0004143 (Reference 34 in the published article) [8]

Butler RK, Finn DP (2009) Stress-induced analgesia. Prog Neurobiol 88: 184–202. doi: 10.1016/j.pneurobio.2009.04.003 (Reference 18 in the published article) [9]

Institute for Laboratory Animal Research (2009) Recognition and alleviation of pain in laboratory animals. In: Recognition and Assessment of Pain. Washington, D.C: National Academies Press. pp. 47–70. (Reference 16 in the published article) [10]

Spooner MF, Robichaud P, Carrier JC, Marchand S (2007) Endogenous pain modulation during the formalin test in estrogen receptor beta knockout mice. Neuroscience 150: 675–680. doi: 10.1016/j.neuroscience.2007.09.037 (Reference 40 in the published article) [11]

Borsook TK, MacDonald G (2010) Mildly negative social encounters reduce physical pain sensitivity. Pain 151: 372–377. doi: 10.1016/j.pain.2010.07.022 (Reference 42 in the published article) [12]

Ford GK, Finn DP (2008) Clinical correlates of stress-induced analgesia: evidence from pharmacological studies. Pain 140: 3–7. doi: 10.1016/j.pain.2008.09.023 (Reference 38 in the published article) [13]

Cachexia in chronic kidney disease: malnutrition-inflammation complex and reverse epidemiology. 2006. DOI 10.1007/978-88-470-0552-5_31 (uncited in the published article) [14]

Leukocytes in the regulation of pain and analgesia. 2005. doi: 10.1189/jlb.0405223 (uncited in the published article) [15]

Sternberg WF (1999) Sex differences in the effects of prenatal stress on stress-induced analgesia. Physiol Behav 68: 63–72. doi: 10.1016/s0031-9384(99)00164-x (Reference 22 in the published article) [16]

Klatzkin RR, Mechlin B, Girdler SS (2010) Menstrual cycle phase does not influence gender differences in experimental pain sensitivity. Eur J Pain 14: 77–82. doi: 10.1016/j.ejpain.2009.01.002 (Reference 57 in the published article) [17]

Pain Perception during Menstrual Cycle. 2011. DOI 10.1007/s11916-011-0207-1 (uncited in the published article) [18]

In view of the extensive verbatim use of text from other sources, the PLOS ONE Editors retract this article.

The authors wish to acknowledge that such re-use of text is not appropriate and would like to apologize.
